# Significance of accurate hilar and intrapulmonary lymph node examination and prognostication in stage IA–IIA non-small cell lung cancer, a retrospective cohort study

**DOI:** 10.1186/s12957-020-02027-y

**Published:** 2020-09-30

**Authors:** Wenyu Zhai, Fangfang Duan, Yuzhen Zheng, Qihang Yan, Shuqin Dai, Tao Chen, Jianlong Chen, Junye Wang

**Affiliations:** 1 Department of Thoracic Surgery, State Key Laboratory of Oncology in South China, Collaborative Innovation Center for Cancer Medicine, Sun Yat-sen University Cancer Center, Guangzhou, Guangdong People’s Republic of China; 2 VIP Region, State Key Laboratory of Oncology in South China, Collaborative Innovation Center for Cancer Medicine, Sun Yat-sen University Cancer Center, Guangzhou, Guangdong People’s Republic of China; 3 Department of Thoracic Surgery, Sun Yat-sen University Sixth Affiliated Hospital, Guangzhou, Guangdong People’s Republic of China; 4 Department of Laboratory Medicine, State Key Laboratory of Oncology in South China, Collaborative Innovation Center for Cancer Medicine, Sun Yat-sen University Cancer Center, Guangzhou, Guangdong People’s Republic of China; 5 Department of Thoracic Surgery, The People’s Hospital of Chao Zhou, Chao Zhou, Guangdong People’s Republic of China; 6 Department of Thoracic Surgery, The Second People’s Hospital of Shan Tou, Shan Tou, Guangdong People’s Republic of China

**Keywords:** Non-small cell lung cancer, Lymph node, Prognosis

## Abstract

**Background:**

The examination of lymph nodes (LNs) plays an important role in the nodal staging of non-small cell lung cancer (NSCLC). For patients without LN metastasis, the main role of thorough LN examination is accurate staging, which weakens the effect of staging migration. To date, the role of hilar and intrapulmonary (N1) station LNs has not been fully appreciated. In this study, we aimed to confirm the significance of N1 LNs in long-term survival for stage IA–IIA NSCLC patients and to find the minimum number of LN to examine.

**Methods:**

The data of patients who underwent radical lobectomy and were confirmed as having non-metastatic LNs from January 2008 to March 2018 were retrospectively screened. Pathology records were reviewed for the number of LNs examined. The Kaplan-Meier method and Cox regression model were used to identify survival and prognostic factors.

**Results:**

The median number of resected N1 LNs was 8. The number of patients with 0–2 N1 LNs, 3–5 N1 LNs, 6–8 N1 LNs, 9–11 N1 LNs, and more than 11 N1 LNs examined was 181, 425, 477, 414, and 531, respectively. Sex (*P* = 0.004), age (*P* < 0.001), tumor size (*P* = 0.004), differentiation degree (*P* = 0.001), and number of N1 LNs examined (*P* = 0.008) were independent prognostic factors of overall survival. Gender (*P* = 0.006), age (*P* = 0.031), tumor size (*P* = 0.001), differentiation degree (*P* = 0.001), vascular invasion (*P* = 0.034), and number of N1 LNs examined (*P* = 0.007) were independent prognostic factors of disease-free survival. Compared with patients with 0–5 N1 LNs examined, patients with more than 5 N1 LNs examined had better OS (*P* = 0.015) and had better DFS (*P* = 0.015) if only a landmark 5-year follow-up was performed.

**Conclusion:**

Increasing the number of N1 LN examination might improve the long-term survival of T1-2N0 NSCLC patients. These data indicate that at least 6 N1 nodes examined is an essential part in surgical and pathological management but cannot prove this. This finding should be confirmed in a large, prospective randomized clinical study.

## Background

Lung cancer remains the most common type of cancer worldwide [1]. For patients with stage IA–IIA non-small cell lung cancer (NSCLC), lobectomy with systemic lymph node (LN) dissection or systematic LN sampling remains the standard therapy [2, 3]. The 5-year overall survival (OS) rate of patients with stages IA–IIA is about 60–92% [4].

Despite radical resection, patients still have a high risk of recurrence. Kelsey et al. reported that the 5-year actuarial risk of disease recurrence was 36% in patients with stage I–II NSCLC [5]. Inadequate LN examination may be an important risk factor for recurrence [6]. The examination of LNs plays an important role in accurate node staging, and node-positive patients need adjuvant therapy to reduce their risk of recurrence [7]. For node-positive patients, the removal of metastatic LNs has a therapeutic effect. However, for node-negative patients, the main role of thorough LN examination is accurate staging, which weakens the effect of staging migration. The so-called staging migration refers to the insufficient LN examination to make LN-positive staging migrate to LN-negative staging. There is still no consensus regarding the optimal number of perioperative LNs to be retrieved for pathological examination. Some studies have shown that a minimum of 10 LNs should be examined to guarantee an accurate N stage [8, 9]. Liang et al. mentioned at least 16 LNs should be removed during the surgical procedure [10]. For mediastinal (N2) station LNs, the debate is mainly concentrated on the selection between systemic mediastinal LN dissection and selective mediastinal LN sampling. Several researchers believe that mediastinal LN dissection should be routinely performed during surgeries [7, 11]. In contrary, some studies revealed that selective mediastinal LN sampling had a similar impact on surgical outcomes compared with systemic mediastinal LN dissection for early NSCLC [12–14]. Currently, few studies have focused on the examination of intrapulmonary or hilar (N1) station LNs. To assess the effect of N1 LN examination on the survival of stages IA–IIA NSCLC patients, we retrospectively screened 3014 NSCLC patients from our center and found the minimum number of N1 LNs to examine.

## Methods

### Patients

This study was approved by the Institutional Review Board of Sun Yat-sen University Cancer Center (SYSUCC). The data of patients who underwent radical surgery at the Thoracic Surgery Department of SYSUCC between January 2008 and March 2018 were reviewed. The inclusion criteria included the following: (1) confirmed pathological diagnosis of NSCLC, (2) pathologically staged as IA–IIA (T1a-2BN0M0), and (3) confirmed negative surgical margin (R0). The exclusion criteria included the following: (1) underwent neoadjuvant therapy, (2) had multiple primary cancers, (3) underwent sublobectomy, and (4) the overall survival (OS) and disease-free survival (DFS) time of less than 1 month. All patients were restaged according to the TNM staging criteria of the 8th National Comprehensive Cancer Network (NCCN) staging system.

### Definition and examination of N1 LNs

In this study, the dissection of N1 and N2 LN stations was performed by surgeons and reconfirmed by pathologists. N1 LNs specifically refer to LNs in the lobe in which the tumor is located. N2 and hilar LNs were dissected by surgeons during the operation. Interlobar, lobar, segmental, and subsegmental LNs were extracorporeally dissected from lung specimens by surgeons after surgery. The information on the number of LNs examined and other pathological factors were reviewed from pathology reports.

### Follow-up

Follow-ups were performed every 3 months in the first 2 years, every 6 months until 5 years, and once a year thereafter. Surgeons would prescribe chest radiography, ultrasonography, or computed tomography (CT) scans as needed during follow-up. The study endpoints were OS and DFS, which were defined as the time from surgery to death and the time from surgery to first the locoregional or distant recurrence or death, respectively.

### Statistical analysis

All statistical analyses were performed using the SPSS software, version 22.0 (SPSS, Inc., Chicago, IL). Analysis of variance (ANOVA) was performed to compare quantitative data and Pearson’s χ^2^, or Kruskal-Wallis *H* test was used to compare categorical data between five groups. The OS and DFS were estimated by using the method of Kaplan-Meier method and compared using the log-rank test. Univariate and multivariate Cox proportional hazards regression analyses were used to identify prognostic factors for survival. The variables assessed in this study included age, gender, tumor size, smoking history, 8th TNM stage, histology, differentiation degree, visceral pleural invasion, vascular invasion, adjuvant chemotherapy, number of N2 LNs examined, number of N1 LNs examined, and the thoracotomy or video-assisted thoracoscopic surgery (VATS). The number of N2 LNs examined was treated as rank variable categorized into 6 groups, namely, 0 to 2, 3 to 5, 6 to 8, 9 to 11, 12 to 15, and more than 15. The number of N1 LNs examined was also treated as rank variable categorized into 5 groups, namely, 0 to 2, 3 to 5, 6 to 8, 9 to 11, and more than 11. Variables whose *P* values were < 0.1 in univariate analyses were included in multivariate analyses. *P* values < 0.05 were considered statistically significant, and all hypotheses were two-sided.

## Results

### Patient characteristics

**Fig. 1:**
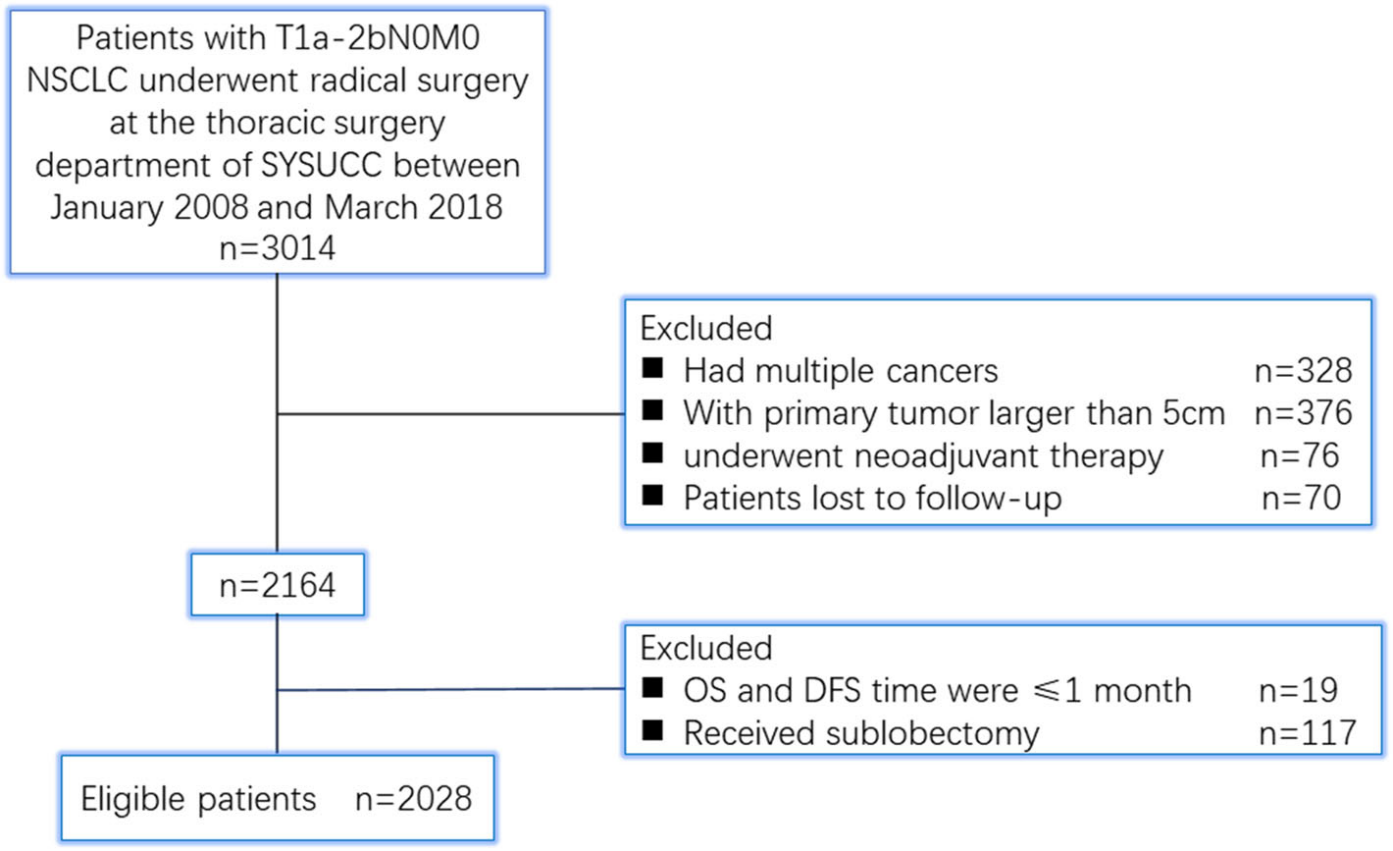
Flowchart of this study

A total of 2028 patients were eligible for analysis (Fig. 1). The patient characteristics are shown in Table 1. The median age of the 1207 male and 812 female patients investigated was 61 years (range, 20 to 83 years). The main pathological type was adenocarcinoma (*n* = 1567). A total of 266 patients received adjuvant chemotherapy. More than half of the patients (*n* = 1101) underwent minimally invasive VATS, and 927 patients underwent traditional thoracotomy. The median number of N2 LNs examined was 11 (range, 0 to 104). The median number of N1 LNs examined was 8 (range, 0 to 38). The number of patients with 0–2, 3–5, 6–8, 9–11, and > 11 examined N1 LNs was 181 (8.9%), 425 (21.0%), 477 (23.5%), 414 (20.4%), and 531(26.2%), respectively.

**Table 1:** Patients characteristics

Characteristics	N1 LN subgroups						*P* value
	0–2	3–5	6–8	9–11	≥ 12	Overall	
	*n* = 181	*n* = 425	*n* = 477	*n* = 414	*n* = 531	*n* = 2028	
Gender							0.289
Male	103 (56.9)	246 (57.9)	276 (57.9)	245 (59.2)	337 (63.5)	1207 (59.4)	
Female	78 (43.1)	179 (42.1)	201 (42.1)	169 (40.8)	194 (36.5)	821 (40.5)	
Age (year)	60.9 ± 9.6	60.3 ± 9.5	59.5 ± 9.8	59.6 ± 9.3	59.8 ± 9.0	59.9 ± 9.4	0.460
Tumor size (cm)	2.4 ± 1.0	2.5 ± 1.0	2.5 ± 1.0	2.6 ± 1.0	2.6 ± 1.0	2.5 ± 1.0	0.027
Smoking history							0.008
No	115 (63.5)	238 (56.0)	268 (56.2)	224 (54.1)	259 (48.8)	1104 (54.4)	
Yes or ever	66 (36.5)	187 (44.0)	209 (43.8)	190 (45.9)	272 (51.2)	924 (45.6)	
8th TNM stage							0.537
IA	86 (47.5)	222 (52.2)	240 (50.3)	194 (46.9)	256 (48.2)	998 (49.2)	
IB–IIA	95 (52.5)	203 (47.8)	237 (49.7)	220 (53.1)	275 (51.8)	1030 (50.8)	
Histology							0.582
Adenocarcinoma	145 (80.1)	329 (77.4)	365 (76.5)	312 (75.4)	416 (78.3)	1567 (77.3)	
Squamous cell carcinoma	19 (10.5)	66 (15.5)	74 (15.5)	68 (16.4)	83 (15.6)	310 (15.3)	
Others	17 (9.4)	30 (7.1)	38 (8.0)	34 (8.2)	32 (6.0)	151 (7.4)	
Differentiation degree							0.054
Well	26 (14.4)	48 (11.3)	28 (5.9)	38 (9.2)	56 (10.5)	196 (9.7)	
Moderate	98 (54.4)	224 (52.8)	275 (57.9)	246 (59.6)	264 (49.7)	1107 (54.7)	
Poor	57 (31.2)	153 (35.9)	174 (36.2)	130 (31.2)	211 (39.7)	725 (35.6)	
Visceral pleural invasion							0.605
Negative	119 (65.7)	306 (72.0)	329 (69.0)	288 (69.6)	375 (70.6)	1417 (69.9)	
Positive	62 (34.3)	119 (28.0)	148 (31.0)	126 (30.4)	156 (29.4)	611 (30.1)	
Vascular invasion							0.843
Negative	168 (92.8)	399 (93.9)	442 (92.7)	391 (94.4)	496 (93.4)	1896 (93.5)	
Positive	13 (7.2)	26 (6.1)	35 (7.3)	23 (5.6)	35 (6.6)	132 (6.5)	
Number of N2 LNs examined							< 0.001
0–2	24 (13.3)	24 (5.6)	29 (6.1)	13 (3.1)	21 (4.0)	111 (5.5)	
3–5	29 (16.0)	62 (14.6)	66 (13.8)	54 (13.0)	53 (10.0)	264 (13.0)	
6–8	40 (22.1)	79 (18.6)	83 (17.4)	68 (16.4)	78 (14.7)	348 (17.2)	
9–11	25 (13.8)	56 (13.2)	83 (17.4)	60 (14.5)	76 (14.3)	300 (14.8)	
12–14	20 (11.0)	72 (16.9)	69 (14.5)	65 (15.7)	72 (13.6)	298 (14.7)	
≥ 15	43 (23.8)	132 (31.1)	147 (30.8)	154 (37.2)	231 (43.5)	707 (34.9)	
Adjuvant chemotherapy							0.582
No	160 (88.4)	363 (85.4)	413 (86.6)	368 (88.9)	458 (48.2)	1762 (86.9)	
Yes or ever	21 (11.6)	62 (14.6)	64 (13.4)	46 (11.1)	73 (13.7)	266 (13.1)	
Thoracotomy or VATS							0.176
Thoracotomy	68 (37.6)	193 (45.4)	230 (48.2)	194 (46.9)	242 (45.6)	927 (45.7)	
VATS	113 (62.4)	232 (54.6)	247 (51.8)	220 (53.1)	289 (54.4)	1101 (54.3)	

### Survival analysis

**Fig. 2:**
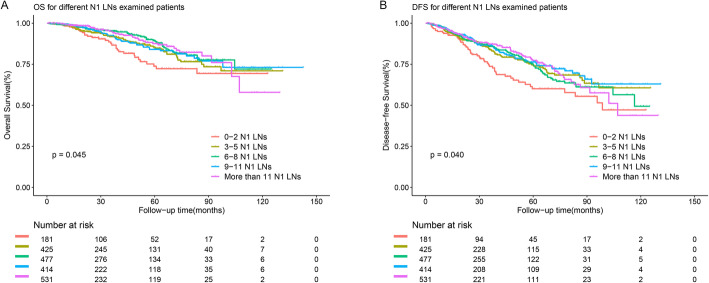
**a** OS for patients with different N1 LNs examined patients. **b** DFS for patients with different N1 LNs examined patients

The 5-year OS rates of patients with 0–2, 3–5, 6–8, 9–11, and > 11 examined N1 LNs were 73.8%, 85.4%, 89.4%, 84.0%, and 87.8%, respectively. The 5-year DFS rates of patients with 0–2, 3–5, 6–8, 9–11, and > 11 examined N1 LNs were 60.1%, 74.6%, 75.1%, 76.4%, and 77.3%, respectively. As shown in Fig. 2, patients with 0–2, 3–5, 6–8, 9–11, and > 11 examined N1 LNs had clearly different OS (log-rank *p* = 0.045, Fig. 2a) and DFS rates (log-rank *p* = 0.045, Fig. 2b).

As shown in Table 2, age, gender, tumor size, smoking history, 8th TNM stage, differentiation degree, vascular invasion, and number of N1 LNs examined were statistically significant in univariate analysis of OS, and visceral pleural invasion also had a *p* value less than 0.1. In multivariate analysis, advanced age (*P* < 0.001; hazard ratio (HR) 1.042; 95% confidence interval (CI) 1.026–1.059), larger tumor size (*P* = 0.004; HR 1.280; 95% CI 1.081–1.516), and differentiation degree (*P* = 0.001; HR 1.350; 95% CI 1.064–1.714) were negatively correlated with OS. Male sex (*P* = 0.004; HR 0.535; 95% CI 0.351–0.814) and number of N1 LNs examined (*P* = 0.007) were positively correlated with OS. Patients with > 11 N1 LNs (*P* < 0.001; HR 0.427; 95% CI 0.266–0.688) and 6–8 N1 LNs (*P* = 0.002; HR 0.484; 95% CI 0.304–0.770) examined were the two groups with the lowest HR value. All factors except adjuvant chemotherapy, number of N2 LNs examined, and thoracotomy or VATS had a *P* value less than 0.1 in the univariate analysis of DFS and were enrolled in multivariate analysis (Table 3). In multivariate analysis, advanced age (*P* = 0.031; HR 1.012; 95% CI 1.001–1.023), larger tumor size (*P* = 0.001; HR 1.237; 95% CI 1.094–1.399), differentiation degree (*P* = 0.001; HR 1.331; 95% CI 1.119–1.584), and vascular invasion (*P* = 0.034; HR 1.506; 95% CI 1.031–2.200) were negatively correlated with DFS. Male sex (*P* = 0.006; HR 0.663; 95% CI 0.495–0.888) and the number of N1 LNs examined were positively correlated with DFS. Patients with > 11 N1 LNs (*P* = 0.001; HR 0.542; 95% CI 0.384–0.766) and 9–11 N1 LNs (*P* = 0.002; HR 0.570; 95% CI 0.399–0.813) examined were the two groups with the lowest HR value.

**Table 2:** Univariate and multivariate analysis of OS

Factors	Univariate analysis		Multivariate Analysis	
	HR (95% CI)	*P* value	HR (95% CI)	*P* value
Gender	0.393 (0.283–0.546)	< 0.001	0.538 (0.352–0.821)	0.004
Age (year)	1.049 (1.033–1.066)	< 0.001	1.042 (1.025–1.058)	< 0.001
Tumor size (cm)	1.461 (1.288–1.657)	< 0.001	1.274 (1.075–1.510)	0.005
Smoking history	2.067 (1.558–2.742)	< 0.001	1.107 (0.764–1.603)	0.592
8th TNM stage	1.818 (1.364–2.425)	< 0.001	1.196 (0.762–1.878)	0.437
Visceral pleural invasion	1.281 (0.973–1.687)	0.077	1.180 (0.818–1.702)	0.377
Histology				
Squamous cell carcinoma	Ref		Ref	
Adenocarcinoma	0.629 (0.459–0.864)	0.004	1.061 (0.745–1.513)	0.742
Others	0,946 (0.581–1.1542)	0.825	1.225 (0.734–2.043)	0.437
Differentiation degree	1.683 (1.341–2.112)	< 0.001	1.349 (1.063–1.711)	0.014
Vascular invasion	1.899 (1.119–3.222)	0.017	1.398 (0.805–2.429)	0.235
Number of N2 LNs examined				
0–2	Ref			
3–5	0.427 (0.244–0.783)	0.006	0.565 (0.302–1.056)	0.073
6–8	0.629 (0.368–1.073)	0.089	0.745 (0.429–1.294)	0.296
9–11	0.516 (0.292–0.913)	0.023	0.663 (0.367–1.198)	0.174
12–14	0.648 (0.372–1.129)	0.126	0.887 (0.497–1.583)	0.686
> 14	0.563 (0.342–0.929)	0.024	0.654 (0.388–1.102)	0.111
Adjuvant chemotherapy	0.925 (0.625–1.369)	0.695		
Thoracotomy or VATS	0.864 (0.655–1.141)	0.304		
Number of N1 LNs examined				
0–2	Ref		Ref	
3–5	0.702 (0.451–1.091)	0.116	0.631 (0.403–0.987)	0.044
6–8	0.533 (0.336–0.844)	0.007	0.489 (0.307–0.779)	0.003
9–11	0.630 (0.398–0.997)	0.049	0.599 (0.375–0.957)	0.032
> 11	0.527 (0.329–1.845)	0.008	0.434 (0.269–0.701)	0.001

**Table 3:** Univariate and multivariate analysis of DFS

Factors	Univariate analysis		Multivariate analysis	
	HR (95% CI)	*P* value	HR (95% CI)	*P* value
Gender	0.570 (0.458–0.709)	< 0.001	0.665 (0.496–0.892)	0.006
Age (year)	1.018 (1.007–1.029)	0.002	1.012 (1.001–1.023)	0.039
Tumor size (cm)	1.402 (1.278–1.538)	< 0.001	1.232 (1.089–1.394)	0.001
Smoking history	1.537 (1.258–1.877)	< 0.001	1.026 (0.780–1.348)	0.856
8^th^ TNM stage	1.938 (1.570–2.393)	< 0.001	1.386 (0.997–1.928)	0.052
Visceral pleural invasion	1.333 (1.088–1.631)	0.005	1.053 (0.799–1.373)	0.709
Histology				
Squamous cell carcinoma	Ref		Ref	
Adenocarcinoma	0.782 (0.611–0.999)	0.049	1.222 (0.926–1.614)	0.157
Others	0.946 (0.666–1.433)	0.905	1.179 (0.789–1.762)	0.422
Differentiation degree	1.559 (1.323–1.838)	< 0.001	1.338 (1.124–1.592)	0.001
Vascular invasion	1.982 (1.378–2.852)	< 0.001	1.474 (1.006–2.160)	0.047
Number of N2 LNs examined				
0–2	Ref			
3–5	0.627 (0.391–1.005)	0.052	0.730 (0.451–1.180)	0.199
6–8	0.841 (0.545–1.297)	0.433	0.916 (0.590–1.423)	0.696
9–11	0.737 (0.470–1.156)	0.183	0.826 (0.522–1.307)	0.413
12–14	0.730 (0.463–1.151)	0.175	0.869 (0.545–1.385)	0.554
> 14	0.759 (0.505–1.142)	0.186	0.665 (0.555–1.285)	0.431
Adjuvant chemotherapy	1.127 (0.858–1.481)	0.500		
Thoracotomy or VATS	0.933 (0.763–1.141)	0.304		
Number of N1 stations examined				
0–2	Ref		Ref	
3–5	0.654 (0.464–0.922)	0.015	0.640 (0.452–0.906)	0.012
6–8	0.680 (0.486–0.949)	0.024	0.658 (0.469–0.924)	0.016
9–11	0.595 (0.418–0.847)	0.004	0.585 (0.407–0.839)	0.004
> 11	0.627 (0.445–0.884)	0.008	0.557 (0.391–0.793)	0.001

**Fig. 3:**
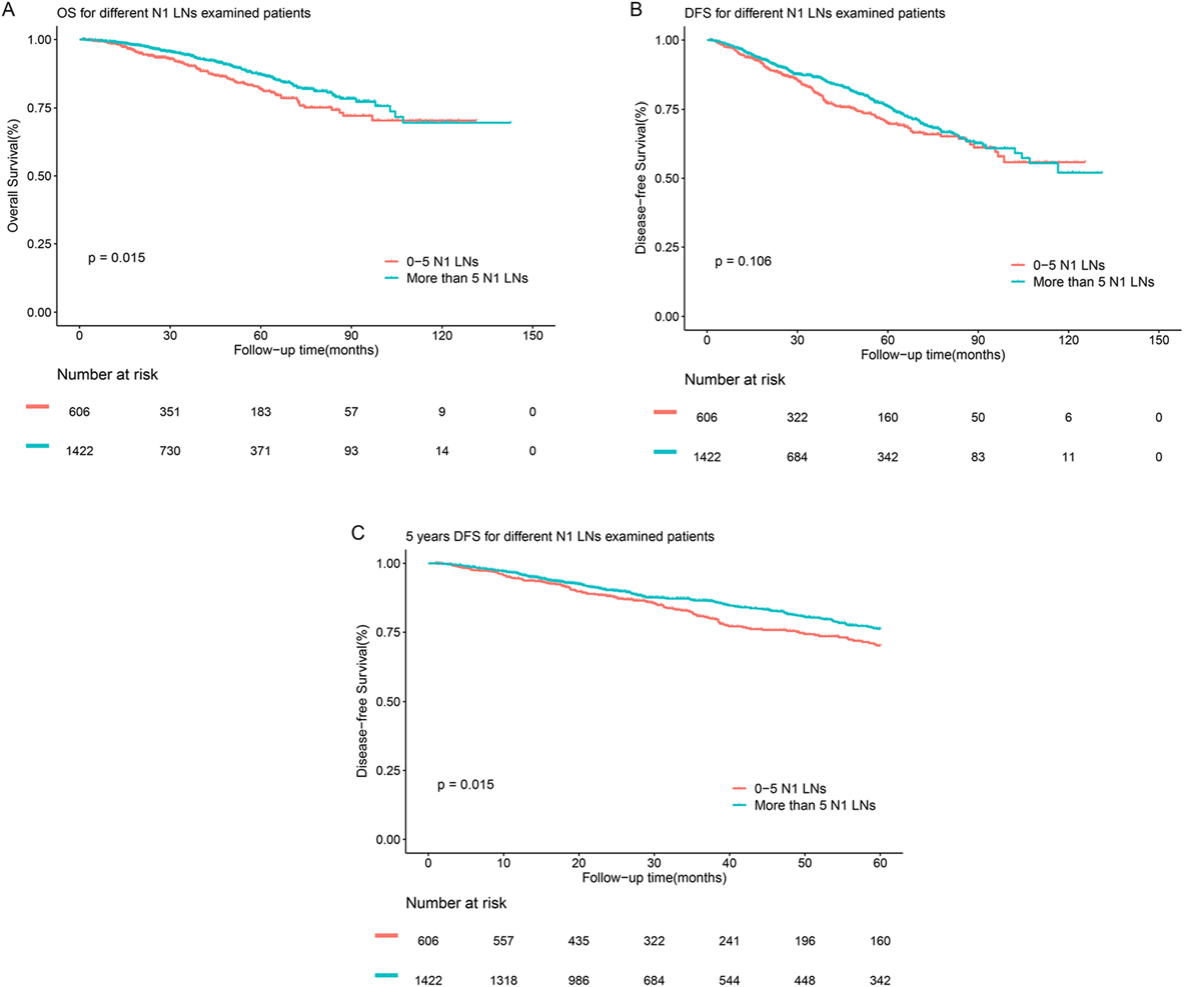
**a** OS for patients with 0–5 and more than 5 N1 LNs examined patients. **b** DFS for patients with 0–5 and more than 5 N1 N1 LNs examined patients. **c** Five-year DFS for patients with 0–5 and more than 5 N1 LNs examined patients

Then, we compared prognosis between patients with 0–5 N1 LNs examined and patients with more than 5 N1 LNs examined. As shown in Fig. 3, the 5-year OS rates of patients with more than 5 N1 LNs examined was significantly higher than the 5-year OS rates of patients with 0–5 N1 LNs examined (87.1% versus 81.9%, log-rank *P* = 0.015, Fig. 3a), while the 5-year DFS rates of patients with more than 5 N1 LNs examined was not apparently higher than the 5-year DFS rates of patients with 0–5 N1 LNs examined (76.2% versus 70.2%, log-rank *p* = 0.106, Fig. 3b). And examining more than 5 N1 LNs is a positive prognostic factor in multivariate analyses of OS (*P* = 0.013; HR 0.704; 95% CI 0.533–0.929) and DFS (*P* = 0.035; HR 0.800; 95% CI 0.650–0.984). If only a landmark 5-year follow-up was performed, patients with more than 5 N1 LNs examined had significantly better DFS (*P* = 0.015, Fig. 3c).

## Discussion

In the treatment of NSCLC patients, accurate node staging is important for predicting prognosis and selecting an appropriate treatment strategy. Clinical N staging based solely on CT and positron emission tomography (PET) is not accurate enough for early NSCLC [15]. Thorough postoperative pathological N examination is optimal for N staging. The number of LNs examined is an intuitive indicator of examination thoroughness. In this study, we found that the number of N1 LNs examined was an independent prognostic factor of OS and DFS for stage IA–IIA patients.

There are some potential explanations for the survival advantage brought by the larger number of N1 LNs examined. An increasing number of N1 LNs examined would lead to a greater probability of discovering metastasized LNs in the hilum and lung, leading to stage migration, which is considered to be the main factor for the improvement of OS and DFS in patients with a large number of N1 LNs examined. Inadequate LN examination may result in some metastatic LNs not being detected, and patients would be wrongly staged as IA or IB when they should be staged as IIB and receive adjuvant therapy. The resection of micrometastases and the effect of the immunologic microenvironment may also be related to the survival advantage brought by the larger number of N1 LNs examined [16–19]. In this study, patients who underwent sublobectomy were excluded which means all intrapulmonary lymph nodes are removed along with the lobes. The resection of micrometastases did not have a significant impact on the survival advantage brought by the larger number of N1 LNs examined.

Several researchers have emphasized that a larger number of LNs examined could increase the accuracy of N staging and enhance prognosis. Samayoa et al. retrospectively analyzed 98,970 patients from The National Cancer Data Base (NCDB) and found that the amounts of LNs examination apparently affect the long-term survival, and at least 10 LNs should be examined in surgical management [17]. Ou and Zell retrospectively investigated the data of 2545 patients and confirmed that the number of LN examination was the favorable prognostic factor for stage IA patients and suggested that the removal of 11–15 LNs could improve the patients prognosis [20]. However, the above two studies did not separately analyze the effects of N1 and N2 LNs on survival.

In our pilot study, the number of LNs examined was identified as an independent prognostic factor for OS (*P* = 0.005). In this study, the survival advantage from the increase in the number of LNs examined should be attributed to the increase in the number of N1 LNs examined, which might be associated with the following reason. The patients in this study underwent a high-quality mediastinal LN dissection. The number of patients with at least 1, 2, and 3 N2 stations dissected was 1992 (98.2%), 1864 (91.9%), and 1457 (71.8%), respectively, and the median number of N2 LNs examined was 11 in this study. The median number of N2 LNs examined was 12 in the American College of Surgeons Oncology Group (ACOSOG) Z0030 trial which had a superb quality of N2 LN examination [21]. There is no apparent difference in the median number of N2 LNs examined between this study and the ACOSOG Z0030 trial. As the number of N2 LNs examined increases, the survival advantage decreases when the quality of N2 LN examination increases to a high level. The number of N2 LNs examined lost statistical significance in the Cox regression model. However, this result cannot deny the vital role of N2 LN examination in node staging. Both N1 and N2 LN examinations are important for accurate node staging.

Some researchers have focused on the importance of the number of N1 LNs examined. Saynak et al. reported that T1N0 patients with inadequate N1 LNs examined had similar local recurrence-free survival compared with T1N1 patients [22]. The ACOSOG Z0030 trial also found a tendency that the greater the number of intrapulmonary LNs examined, the better the patient survival outcomes [23]. Varlotto et al. demonstrated that a minimum of 11 to 16 LNs should be examined when examining only N1 lymph nodes [24]. Similar to the finding of the above study, patients with more than 11 N1 LNs examined had the lowest HR values in the multivariate analyses of OS and DFS, signifying that at least 12 N1 LNs should be examined to achieve optimal OS and DFS. However, it is difficult to accomplish this goal in clinical practice. Only 26.2% of patients accepted the examination of more than 11 N1 LNs in this study. On the one hand, patients with 6–8 N1 LNs examined had the second lowest HR values in the multivariate analysis of OS, and patients with more than 5 N1 LNs examined had better OS and DFS in landmark 5-year follow-up. The survival analyses showed examining at least 6 N1 LNs could bring survival advantages. On the other hand, the median number of N1 LNs examined in this study was 8, which means that more than half of the patients did not have at least 9 N1 LNs examined. As a comparison, the median number of N1 LNs examined was 5 in the ACOSOG Z0030 trial and 5 in Saynak et al.’s study. It is not easy to examine at least 9 N1 LNs for each patient. The clinical decision should be feasible, and at least 6 N1 LNs examined is a realistic goal in clinical practice. Therefore, we recommend at least 6 N1 LNs examined in surgical and pathological management.

However, the examination of N1 LNs has not received enough attention. One of the phenomena was that the quality of LN examination exhibits noteworthy variability during surgical and pathological management [25, 26]. Another phenomenon is that incomplete intrapulmonary LNs were retrieved in pathological examination. One previous study revealed that a median of six additional LNs was discovered after rechecking remnant lung specimens, and the median number of N1 LNs examined was only 3 in the community-based Memphis Metropolitan Area Quality of Surgical Resection cohort [27]. Although N2 LN examinations were of superb quality, the median number of N1 LNs examined was 5 in the ACOSOG Z0030 trial [21]. In this study, the median number of resected N1 LNs was eight. The pattern of LN examination in which N1 LNs were dissected by surgeons and reconfirmed by pathologists contributed to this result.

In addition to insufficient attention to N1 LN examination possibly affecting the number of N1 LNs examined, the surgical approach may also affect the number of N1 LNs examined. Subramanian et al. reviewed 1687 patients with stage IA NSCLC from the National Cancer Database, and included 1354 patients who underwent lobectomy and 333 patients who underwent sublobar resections. They found that sublobar resection had inadequate LNs examined and was associated with a 39% increased risk of recurrence. The majority of patients were treated with sublobar resection [28]. For both sublobar resection and lobectomy, the procedure of mediastinal LN dissection does not have apparent differences. However, sublobar resection, especially wedge resection, might lose some N1 LNs, which is associated with poor outcomes. In addition, when we dissected intrapulmonary LNs, we found a phenomenon in which patients whose primary tumor was near a segmental bronchus were prone to segmental LN metastases. Therefore, we suggest that patients whose primary tumor is near a segmental bronchus need a careful intrapulmonary LN dissection. Furthermore, intrapulmonary LNs are dissected extracorporeally, if 6 lymph nodes were not dissected in the first operation, it is safe to dissect the lung specimens again to take an LN sample for accurate staging.

There are some limitations to this study. First, this was a single-center retrospective study and associated biases may have been inevitable, and external validation was not performed to validate the findings. Second, this study lacks incidence of local LN failure which makes “stage migration” more convincing. Although intrapulmonary node was removed with lobe, local LN failure will still be associated with unsatisfied hilar LN dissection. In addition, the data of this study did not find a survival advantage from the increase in the number of N2 LNs examined and cannot answer how many N2 LNs should be examined in surgical and pathological management. Therefore, further validation from multicenter database is needed, and, the findings from this study should be cautiously interpreted.

## Conclusions

Increasing the number of N1 LNs examined might improve the long-term survival of T1-2N0 NSCLC patients. The data of this study indicate that at least 6 N1 nodes examined is an essential part in surgical and pathological management but cannot prove this. This finding should be confirmed in a large, prospective randomized clinical study.

## Data Availability

The key raw data have been deposited into the Research Data Deposit (http://www.researchdata.org.cn), with the approval number of RDDA2020001355, and the datasets used in this study are publicly available.

## Abbreviations

NSCLC: Non-small cell lung cancer
LDCT: Low-dose computed tomography
NCCN: National Comprehensive Cancer Network
LN: Lymph node
OS: Overall survival
DFS: Disease-free survival
ACOSOG: American College of Surgeons Oncology Group
